# Supplementary material for: Resolving the full spectrum of human genome variation using Linked-Reads
Source: Genome Res. 2019 Apr;29(4):635–45. doi: 10.1101/gr.234443.118 (PMC6442396; doi:10.1101/gr.234443.118)
Supplement: Supplemental Material [file supp_gr.234443.118_Supplemental_longranger-2.2.zip › longranger-2.2.tar.bz2!/lr2.2-public/lib/go/src/code.google.com/vitess/docs/getting-started/index.html]

Vitess / Running Vitess on Kubernetes


Toggle navigation

- Overview
- Guides
- Reference
- Blog
- GitHub

- #### Overview

  - What is Vitess
  - Scaling MySQL with Vitess
  - Key Concepts
- #### Getting Started

  - Run Vitess on Kubernetes
    - Custom Docker Build
  - Run Vitess Locally
- #### User Guide

  - Introduction- Client Libraries- Backing Up Data- Reparenting
        - Schema Management
        - VSchema Guide
        - Schema Swap (Tutorial)
        - Sharding
          - Horizontal Sharding (Tutorial, automated)
          - Horizontal Sharding (Tutorial, manual)
          - Sharding in Kubernetes (Tutorial, automated)
          - Sharding in Kubernetes (Tutorial, manual)
        - Vitess Sequences
        - MySQL Server Protocol
        - Vitess and Replication
        - Update Stream
        - Row Based Replication
        - Topology Service
        - Transport Security Model
        - Launching
          - Scalability Philosophy
          - Production Planning
          - Server Configuration
          - 2PC Guide
          - Troubleshooting
        - Upgrading
- #### Reference Guides

  - Vitess API- vtctl Commands
- #### Other Resources

  - Presentations- Blog- Roadmap
- #### Contributing

  - Contributing to Vitess- GitHub Workflow- Code Reviews
- GitHub

# Running Vitess on Kubernetes

- #### Overview

  - What is Vitess
  - Scaling MySQL with Vitess
  - Key Concepts
- #### Getting Started

  - Run Vitess on Kubernetes
    - Custom Docker Build
  - Run Vitess Locally
- #### User Guide

  - Introduction- Client Libraries- Backing Up Data- Reparenting
        - Schema Management
        - VSchema Guide
        - Schema Swap (Tutorial)
        - Sharding
          - Horizontal Sharding (Tutorial, automated)
          - Horizontal Sharding (Tutorial, manual)
          - Sharding in Kubernetes (Tutorial, automated)
          - Sharding in Kubernetes (Tutorial, manual)
        - Vitess Sequences
        - MySQL Server Protocol
        - Vitess and Replication
        - Update Stream
        - Row Based Replication
        - Topology Service
        - Transport Security Model
        - Launching
          - Scalability Philosophy
          - Production Planning
          - Server Configuration
          - 2PC Guide
          - Troubleshooting
        - Upgrading
- #### Reference Guides

  - Vitess API- vtctl Commands
- #### Other Resources

  - Presentations- Blog- Roadmap
- #### Contributing

  - Contributing to Vitess- GitHub Workflow- Code Reviews

This page explains how to run Vitess on Kubernetes.
It also gives the steps to start a Kubernetes cluster with
Google Container Engine.

If you already have Kubernetes v1.0+ running in one of the other
supported platforms,
you can skip the `gcloud` steps.
The `kubectl` steps will apply to any Kubernetes cluster.

## Prerequisites

To complete the exercise in this guide, you must locally install Go 1.8+,
Vitess' `vtctlclient` tool, and Google Cloud SDK. The
following sections explain how to set these up in your environment.

### Install Go 1.8+

You need to install Go 1.8+ to build the
`vtctlclient` tool, which issues commands to Vitess.

After installing Go, make sure your `GOPATH` environment
variable is set to the root of your workspace. The most common setting
is `GOPATH=$HOME/go`, and the value should identify a
directory to which your non-root user has write access.

In addition, make sure that `$GOPATH/bin` is included in
your `$PATH`. More information about setting up a Go
workspace can be found at
How to Write Go Code.

### Build and install vtctlclient

The `vtctlclient` tool issues commands to Vitess.

```
$ go get github.com/youtube/vitess/go/cmd/vtctlclient
```

This command downloads and builds the Vitess source code at:

```
$GOPATH/src/github.com/youtube/vitess/
```

It also copies the built `vtctlclient` binary into `$GOPATH/bin`.

### Set up Google Compute Engine, Container Engine, and Cloud tools

**Note:** If you are running Kubernetes elsewhere, skip to
Locate kubectl.

To run Vitess on Kubernetes using Google Compute Engine (GCE),
you must have a GCE account with billing enabled. The instructions
below explain how to enable billing and how to associate a billing
account with a project in the Google Developers Console.

1. Log in to the Google Developers Console to enable billing.

   1. Click the **Billing** pane if you are not there already.
   2. Click **New billing account**.
   3. Assign a name to the billing account -- e.g. "Vitess on
      Kubernetes." Then click **Continue**. You can sign up
      for the free trial
      to avoid any charges.
2. Create a project in the Google Developers Console that uses
   your billing account:

   1. At the top of the Google Developers Console, click the **Projects** dropdown.
   2. Click the Create a Project... link.
   3. Assign a name to your project. Then click the **Create** button.
      Your project should be created and associated with your
      billing account. (If you have multiple billing accounts,
      confirm that the project is associated with the correct account.)
   4. After creating your project, click **API Manager** in the left menu.
   5. Find **Google Compute Engine** and **Google Container Engine API**.
      (Both should be listed under "Google Cloud APIs".)
      For each, click on it, then click the **"Enable API"** button.
3. Follow the Google Cloud SDK quickstart instructions to set up
   and test the Google Cloud SDK. You will also set your default project
   ID while completing the quickstart.

   **Note:** If you skip the quickstart guide because you've previously set up
   the Google Cloud SDK, just make sure to set a default project ID by running
   the following command. Replace `PROJECT` with the project ID assigned to
   your Google Developers Console
   project. You can find the ID
   by navigating to the **Overview** page for the project in the Console.

   ```
   $ gcloud config set project PROJECT
   ```
4. Install or update the `kubectl` tool:

   ```
   $ gcloud components update kubectl
   ```

### Locate kubectl

Check if `kubectl` is on your `PATH`:

```
$ which kubectl
### example output:
# ~/google-cloud-sdk/bin/kubectl
```

If `kubectl` isn't on your `PATH`, you can tell our scripts where
to find it by setting the `KUBECTL` environment variable:

```
$ export KUBECTL=/example/path/to/google-cloud-sdk/bin/kubectl
```

## Start a Container Engine cluster

**Note:** If you are running Kubernetes elsewhere, skip to
Start a Vitess cluster.

1. Set the zone
   that your installation will use:

   ```
   $ gcloud config set compute/zone us-central1-b
   ```
2. Create a Container Engine cluster:

   ```
   $ gcloud container clusters create example --machine-type n1-standard-4 --num-nodes 5 --scopes storage-rw
   ### example output:
   # Creating cluster example...done.
   # Created [https://container.googleapis.com/v1/projects/vitess/zones/us-central1-b/clusters/example].
   # kubeconfig entry generated for example.
   ```

   **Note:** The `--scopes storage-rw` argument is necessary to allow
   built-in backup/restore
   to access Google Cloud Storage.
3. Create a Cloud Storage bucket:

   To use the Cloud Storage plugin for built-in backups, first create a
   bucket
   for Vitess backup data. See the
   bucket naming guidelines
   if you're new to Cloud Storage.

   ```
   $ gsutil mb gs://my-backup-bucket
   ```

## Start a Vitess cluster

1. **Navigate to your local Vitess source code**

   This directory would have been created when you installed
   `vtctlclient`:

   ```
   $ cd $GOPATH/src/github.com/youtube/vitess/examples/kubernetes
   ```
2. **Configure site-local settings**

   Run the `configure.sh` script to generate a `config.sh` file, which will be
   used to customize your cluster settings.

   Currently, we have out-of-the-box support for storing
   backups in
   Google Cloud Storage. If you're using
   GCS, fill in the fields requested by the configure script, including the
   name of the bucket you created above.

   ```
   vitess/examples/kubernetes$ ./configure.sh
   ### example output:
   # Backup Storage (file, gcs) [gcs]:
   # Google Developers Console Project [my-project]:
   # Google Cloud Storage bucket for Vitess backups: my-backup-bucket
   # Saving config.sh...
   ```

   For other platforms, you'll need to choose the `file` backup storage plugin,
   and mount a read-write network volume into the `vttablet` and `vtctld` pods.
   For example, you can mount any storage service accessible through NFS into a
   Kubernetes volume.
   Then provide the mount path to the configure script here.

   Direct support for other cloud blob stores like Amazon S3 can be added by
   implementing the Vitess BackupStorage plugin interface.
   Let us know on the discussion forum
   if you have any specific plugin requests.
3. **Start an etcd cluster**

   The Vitess topology service
   stores coordination data for all the servers in a Vitess cluster.
   It can store this data in one of several consistent storage systems.
   In this example, we'll use etcd.
   Note that we need our own etcd clusters, separate from the one used by
   Kubernetes itself.

   ```
   vitess/examples/kubernetes$ ./etcd-up.sh
   ### example output:
   # Creating etcd service for global cell...
   # service "etcd-global" created
   # service "etcd-global-srv" created
   # Creating etcd replicationcontroller for global cell...
   # replicationcontroller "etcd-global" created
   # ...
   ```

   This command creates two clusters. One is for the
   global cell,
   and the other is for a
   local cell
   called *test*. You can check the status of the
   pods
   in the cluster by running:

   ```
   $ kubectl get pods
   ### example output:
   # NAME                READY     STATUS    RESTARTS   AGE
   # etcd-global-8oxzm   1/1       Running   0          1m
   # etcd-global-hcxl6   1/1       Running   0          1m
   # etcd-global-xupzu   1/1       Running   0          1m
   # etcd-test-e2y6o     1/1       Running   0          1m
   # etcd-test-m6wse     1/1       Running   0          1m
   # etcd-test-qajdj     1/1       Running   0          1m
   ```

   It may take a while for each Kubernetes node to download the
   Docker images the first time it needs them. While the images
   are downloading, the pod status will be Pending.

   **Note:** In this example, each script that has a name ending in
   `-up.sh` also has a corresponding `-down.sh`
   script, which can be used to stop certain components of the
   Vitess cluster without bringing down the whole cluster. For
   example, to tear down the `etcd` deployment, run:

   ```
   vitess/examples/kubernetes$ ./etcd-down.sh
   ```
4. **Start vtctld**

   The `vtctld` server provides a web interface to inspect the state of the
   Vitess cluster. It also accepts RPC commands from `vtctlclient` to modify
   the cluster.

   ```
   vitess/examples/kubernetes$ ./vtctld-up.sh
   ### example output:
   # Creating vtctld ClusterIP service...
   # service "vtctld" created
   # Creating vtctld replicationcontroller...
   # replicationcontroller "vtctld" create createdd
   ```
5. **Access vtctld web UI**

   To access vtctld from outside Kubernetes, use kubectl proxy
   to create an authenticated tunnel on your workstation:

   **Note:** The proxy command runs in the foreground,
   so you may want to run it in a separate terminal.

   ```
   $ kubectl proxy --port=8001
   ### example output:
   # Starting to serve on localhost:8001
   ```

   You can then load the vtctld web UI on `localhost`:

   http://localhost:8001/api/v1/proxy/namespaces/default/services/vtctld:web/

   You can also use this proxy to access the Kubernetes Dashboard,
   where you can monitor nodes, pods, and services:

   http://localhost:8001/ui
6. **Use vtctlclient to send commands to vtctld**

   You can now run `vtctlclient` locally to issue commands
   to the `vtctld` service on your Kubernetes cluster.

   To enable RPC access into the Kubernetes cluster, we'll again use
   `kubectl` to set up an authenticated tunnel. Unlike the HTTP proxy
   we used for the web UI, this time we need raw port forwarding
   for vtctld's gRPC port.

   Since the tunnel needs to target a particular vtctld pod name,
   we've provided the `kvtctl.sh` script, which uses `kubectl` to
   discover the pod name and set up the tunnel before running `vtctlclient`.

   Now, running `kvtctl.sh help` will test your connection to
   `vtctld` and also list the `vtctlclient`
   commands that you can use to administer the Vitess cluster.

   ```
   vitess/examples/kubernetes$ ./kvtctl.sh help
   ### example output:
   # Available commands:
   #
   # Tablets:
   #   InitTablet ...
   # ...
   ```

   You can also use the `help` command to get more details about each command:

   ```
   vitess/examples/kubernetes$ ./kvtctl.sh help ListAllTablets
   ```

   See the vtctl reference for a
   web-formatted version of the `vtctl help` output.
7. **Start vttablets**

   A Vitess tablet is the
   unit of scaling for the database. A tablet consists of the
   `vttablet` and `mysqld` processes, running on the same
   host. We enforce this coupling in Kubernetes by putting the respective
   containers for vttablet and mysqld inside a single
   pod.

   Run the following script to launch the vttablet pods, which also include
   mysqld:

   ```
   vitess/examples/kubernetes$ ./vttablet-up.sh
   ### example output:
   # Creating test_keyspace.shard-0 pods in cell test...
   # Creating pod for tablet test-0000000100...
   # pod "vttablet-100" created
   # Creating pod for tablet test-0000000101...
   # pod "vttablet-101" created
   # Creating pod for tablet test-0000000102...
   # pod "vttablet-102" created
   # Creating pod for tablet test-0000000103...
   # pod "vttablet-103" created
   # Creating pod for tablet test-0000000104...
   # pod "vttablet-104" created
   ```

   In the vtctld web UI, you should soon see a
   keyspace named `test_keyspace`
   with a single shard named `0`.
   Click on the shard name to see the list of tablets. When all 5 tablets
   show up on the shard status page, you're ready to continue. Note that it's
   normal for the tablets to be unhealthy at this point, since you haven't
   initialized the databases on them yet.

   It can take some time for the tablets to come up for the first time if a pod
   was scheduled on a node that hasn't downloaded the Vitess Docker image yet. You can also check the status of the
   tablets from the command line using `kvtctl.sh`:

   ```
   vitess/examples/kubernetes$ ./kvtctl.sh ListAllTablets test
   ### example output:
   # test-0000000100 test_keyspace 0 spare 10.64.1.6:15002 10.64.1.6:3306 []
   # test-0000000101 test_keyspace 0 spare 10.64.2.5:15002 10.64.2.5:3306 []
   # test-0000000102 test_keyspace 0 spare 10.64.0.7:15002 10.64.0.7:3306 []
   # test-0000000103 test_keyspace 0 spare 10.64.1.7:15002 10.64.1.7:3306 []
   # test-0000000104 test_keyspace 0 spare 10.64.2.6:15002 10.64.2.6:3306 []
   ```
8. **Initialize MySQL databases**

   Once all the tablets show up, you're ready to initialize the underlying
   MySQL databases.

   **Note:** Many `vtctlclient` commands produce no output on success.

   First, designate one of the tablets to be the initial master. Vitess will
   automatically connect the other slaves' mysqld instances so that they start
   replicating from the master's mysqld. This is also when the default database
   is created. Since our keyspace is named `test_keyspace`, the MySQL database
   will be named `vt_test_keyspace`.

   ```
   vitess/examples/kubernetes$ ./kvtctl.sh InitShardMaster -force test_keyspace/0 test-0000000100
   ### example output:
   # master-elect tablet test-0000000100 is not the shard master, proceeding anyway as -force was used
   # master-elect tablet test-0000000100 is not a master in the shard, proceeding anyway as -force was used
   ```

   **Note:** Since this is the first time the shard has been started, the
   tablets are not already doing any replication, and there is no existing
   master. The `InitShardMaster` command above uses the `-force` flag to bypass
   the usual sanity checks that would apply if this wasn't a brand new shard.

   After the tablets finish updating, you should see one **master**, and
   several **replica** and **rdonly** tablets:

   ```
   vitess/examples/kubernetes$ ./kvtctl.sh ListAllTablets test
   ### example output:
   # test-0000000100 test_keyspace 0 master 10.64.1.6:15002 10.64.1.6:3306 []
   # test-0000000101 test_keyspace 0 replica 10.64.2.5:15002 10.64.2.5:3306 []
   # test-0000000102 test_keyspace 0 replica 10.64.0.7:15002 10.64.0.7:3306 []
   # test-0000000103 test_keyspace 0 rdonly 10.64.1.7:15002 10.64.1.7:3306 []
   # test-0000000104 test_keyspace 0 rdonly 10.64.2.6:15002 10.64.2.6:3306 []
   ```

   The **replica** tablets are used for serving live web traffic, while the
   **rdonly** tablets are used for offline processing, such as batch jobs and backups.
   The amount of each tablet type
   that you launch can be configured in the `vttablet-up.sh` script.
9. **Create a table**

   The `vtctlclient` tool can be used to apply the database schema
   across all tablets in a keyspace. The following command creates
   the table defined in the `create_test_table.sql` file:

   ```
   # Make sure to run this from the examples/kubernetes dir, so it finds the file.
   vitess/examples/kubernetes$ ./kvtctl.sh ApplySchema -sql "$(cat create_test_table.sql)" test_keyspace
   ```

   The SQL to create the table is shown below:

   ```
   CREATE TABLE messages (
     page BIGINT(20) UNSIGNED,
     time_created_ns BIGINT(20) UNSIGNED,
     message VARCHAR(10000),
     PRIMARY KEY (page, time_created_ns)
   ) ENGINE=InnoDB
   ```

   You can run this command to confirm that the schema was created
   properly on a given tablet, where `test-0000000100`
   is a tablet alias as shown by the `ListAllTablets` command:

   ```
   vitess/examples/kubernetes$ ./kvtctl.sh GetSchema test-0000000100
   ### example output:
   # {
   #   "DatabaseSchema": "CREATE DATABASE `` /*!40100 DEFAULT CHARACTER SET utf8 */",
   #   "TableDefinitions": [
   #     {
   #       "Name": "messages",
   #       "Schema": "CREATE TABLE `messages` (\n  `page` bigint(20) unsigned NOT NULL DEFAULT '0',\n  `time_created_ns` bigint(20) unsigned NOT NULL DEFAULT '0',\n  `message` varchar(10000) DEFAULT NULL,\n  PRIMARY KEY (`page`,`time_created_ns`)\n) ENGINE=InnoDB DEFAULT CHARSET=utf8",
   #       "Columns": [
   #         "page",
   #         "time_created_ns",
   #         "message"
   #       ],
   # ...
   ```
10. **Take a backup**

    Now that the initial schema is applied, it's a good time to take the first
    backup. This backup
    will be used to automatically restore any additional replicas that you run,
    before they connect themselves to the master and catch up on replication.
    If an existing tablet goes down and comes back up without its data, it will
    also automatically restore from the latest backup and then resume replication.

    Select one of the **rdonly** tablets and tell it to take a backup. We use a
    **rdonly** tablet instead of a **replica** because the tablet will pause
    replication and stop serving during data copy to create a consistent snapshot.

    ```
    vitess/examples/kubernetes$ ./kvtctl.sh Backup test-0000000104
    ```

    After the backup completes, you can list available backups for the shard:

    ```
    vitess/examples/kubernetes$ ./kvtctl.sh ListBackups test_keyspace/0
    ### example output:
    # 2015-10-21.042940.test-0000000104
    ```
11. **Initialize Vitess Routing Schema**

    In the examples, we are just using a single database with no specific
    configuration. So we just need to make that (empty) configuration visible
    for serving. This is done by running the following command:

    ```
    vitess/examples/kubernetes$ ./kvtctl.sh RebuildVSchemaGraph
    ```

    (As it works, this command will not display any output.)
12. **Start vtgate**

    Vitess uses vtgate to route each client
    query to the correct `vttablet`. In Kubernetes, a `vtgate` service
    distributes connections to a pool of `vtgate` pods. The pods are curated by
    a replication controller.

    ```
    vitess/examples/kubernetes$ ./vtgate-up.sh
    ### example output:
    # Creating vtgate service in cell test...
    # service "vtgate-test" created
    # Creating vtgate replicationcontroller in cell test...
    # replicationcontroller "vtgate-test" created
    ```

## Test your cluster with a client app

The GuestBook app in the example is ported from the
Kubernetes GuestBook example.
The server-side code has been rewritten in Python to use Vitess as the storage
engine. The client-side code (HTML/JavaScript) has been modified to support
multiple Guestbook pages, which will be useful to demonstrate Vitess sharding in
a later guide.

```
vitess/examples/kubernetes$ ./guestbook-up.sh
### example output:
# Creating guestbook service...
# services "guestbook" created
# Creating guestbook replicationcontroller...
# replicationcontroller "guestbook" created
```

As with the `vtctld` service, by default the GuestBook app is not accessible
from outside Kubernetes. In this case, since this is a user-facing frontend,
we set `type: LoadBalancer` in the GuestBook service definition,
which tells Kubernetes to create a public
load balancer
using the API for whatever platform your Kubernetes cluster is in.

You also need to allow access through your platform's firewall.

```
# For example, to open port 80 in the GCE firewall:
$ gcloud compute firewall-rules create guestbook --allow tcp:80
```

**Note:** For simplicity, the firewall rule above opens the port on **all**
GCE instances in your project. In a production system, you would likely
limit it to specific instances.

Then, get the external IP of the load balancer for the GuestBook service:

```
$ kubectl get service guestbook
### example output:
# NAME        CLUSTER-IP      EXTERNAL-IP     PORT(S)   AGE
# guestbook   10.67.242.247   3.4.5.6         80/TCP    1m
```

If the `EXTERNAL-IP` is still empty, give it a few minutes to create
the external load balancer and check again.

Once the pods are running, the GuestBook app should be accessible
from the load balancer's external IP. In the example above, it would be at
`http://3.4.5.6`.

You can see Vitess' replication capabilities by opening the app in
multiple browser windows, with the same Guestbook page number.
Each new entry is committed to the master database.
In the meantime, JavaScript on the page continuously polls
the app server to retrieve a list of GuestBook entries. The app serves
read-only requests by querying Vitess in 'replica' mode, confirming
that replication is working.

You can also inspect the data stored by the app:

```
vitess/examples/kubernetes$ ./kvtctl.sh ExecuteFetchAsDba test-0000000100 "SELECT * FROM messages"
### example output:
# +------+---------------------+---------+
# | page |   time_created_ns   | message |
# +------+---------------------+---------+
# |   42 | 1460771336286560000 | Hello   |
# +------+---------------------+---------+
```

The GuestBook source code
provides more detail about how the app server interacts with Vitess.

## Try Vitess resharding

Now that you have a full Vitess stack running, you may want to go on to the
Sharding in Kubernetes workflow guide
or Sharding in Kubernetes codelab
(if you prefer to run each step manually through commands) to try out
dynamic resharding.

If so, you can skip the tear-down since the sharding guide picks up right here.
If not, continue to the clean-up steps below.

## Tear down and clean up

Before stopping the Container Engine cluster, you should tear down the Vitess
services. Kubernetes will then take care of cleaning up any entities it created
for those services, like external load balancers.

```
vitess/examples/kubernetes$ ./guestbook-down.sh
vitess/examples/kubernetes$ ./vtgate-down.sh
vitess/examples/kubernetes$ ./vttablet-down.sh
vitess/examples/kubernetes$ ./vtctld-down.sh
vitess/examples/kubernetes$ ./etcd-down.sh
```

Then tear down the Container Engine cluster itself, which will stop the virtual
machines running on Compute Engine:

```
$ gcloud container clusters delete example
```

It's also a good idea to remove any firewall rules you created, unless you plan
to use them again soon:

```
$ gcloud compute firewall-rules delete guestbook
```

## Troubleshooting

### Server logs

If a pod enters the `Running` state, but the server
doesn't respond as expected, use the `kubectl logs`
command to check the pod output:

```
# show logs for container 'vttablet' within pod 'vttablet-100'
$ kubectl logs vttablet-100 vttablet

# show logs for container 'mysql' within pod 'vttablet-100'
# Note that this is NOT MySQL error log.
$ kubectl logs vttablet-100 mysql
```

Post the logs somewhere and send a link to the Vitess
mailing list
to get more help.

### Shell access

If you want to poke around inside a container, you can use `kubectl exec` to run
a shell.

For example, to launch a shell inside the `vttablet` container of the
`vttablet-100` pod:

```
$ kubectl exec vttablet-100 -c vttablet -t -i -- bash -il
root@vttablet-100:/# ls /vt/vtdataroot/vt_0000000100
### example output:
# bin-logs   innodb                  my.cnf      relay-logs
# data       memcache.sock764383635  mysql.pid   slow-query.log
# error.log  multi-master.info       mysql.sock  tmp
```

### Root certificates

If you see in the logs a message like this:

```
x509: failed to load system roots and no roots provided
```

It usually means that your Kubernetes nodes are running a host OS
that puts root certificates in a different place than our configuration
expects by default (for example, Fedora). See the comments in the
etcd controller template
for examples of how to set the right location for your host OS.
You'll also need to adjust the same certificate path settings in the
`vtctld` and `vttablet` templates.

### Status pages for vttablets

Each `vttablet` serves a set of HTML status pages on its primary port.
The `vtctld` interface provides a **STATUS** link for each tablet.

If you access the vtctld web UI through the kubectl proxy as described above,
it will automatically link to the vttablets through that same proxy,
giving you access from outside the cluster.

You can also use the proxy to go directly to a tablet. For example,
to see the status page for the tablet with ID `100`, you could navigate to:

http://localhost:8001/api/v1/proxy/namespaces/default/pods/vttablet-100:15002/debug/status

### Direct connection to mysqld

Since the `mysqld` within the `vttablet` pod is only meant to be accessed
via vttablet, our default bootstrap settings only allow connections from
localhost.

If you want to check or manipulate the underlying mysqld, you can issue
simple queries or commands through `vtctlclient` like this:

```
# Send a query to tablet 100 in cell 'test'.
vitess/examples/kubernetes$ ./kvtctl.sh ExecuteFetchAsDba test-0000000100 "SELECT VERSION()"
### example output:
# +------------+
# | VERSION()  |
# +------------+
# | 5.7.13-log |
# +------------+
```

If you need a truly direct connection to mysqld, you can launch a shell inside the mysql container, and then connect with the `mysql`
command-line client:

```
$ kubectl exec vttablet-100 -c mysql -t -i -- bash -il
root@vttablet-100:/# export TERM=ansi
root@vttablet-100:/# mysql -S /vt/vtdataroot/vt_0000000100/mysql.sock -u vt_dba
```

Contact: vitess@googlegroups.com  **·**  
Announcements  **·**  
© 2017 Vitess powered by Google Inc
